# Film Pictures of Normal Labour and Delivery

**Published:** 1919-11-15

**Authors:** 


### THE CINEMA IN OBSTETRICS.

### Film Pictures of Normal Labour and Delivery.

ON November 11, before the Gynæcological Section of the Royal Society of Medicine, Dr. Drummond Robinson, of Westminster Hospital, successfully demonstrated the possibilities of the cinema as an aid to obstetric teaching, and enabled those present to see the complete process of normal labour represented upon a screen. The film, of about 1,500 feet, was composed of a series of "animated diagrams," graphically representing intra-uterine and pelvic phenomena not otherwise visible, and of actual cinematograph pictures of normal delivery in the second and third stages.

The innovation was most favourably received, and any serious criticism that was possible was softened in the acknowledgment of Dr. Robinson's pioneer labours in thus adapting to teaching purposes a method of demonstration hitherto associated with amusement.

### EDUCATION AND SCIENTIFIC FILMS.

Messrs. Pathé Frères have for many years devoted much time and money to a praiseworthy effort to produce films of an educative and scientific nature; they have in stock some fifty films dealing entirely with medical subjects. Enterprising surgeons have also allowed films of their operative procedures to be made and shown, but it is probably correct to state that this is the first time the cinema has been used to make pictures for the teaching of midwifery. In all the English universities there is but one boasting of a cinematographic installation, and this owes its presence to the generosity of Professor Hall, of Sheffield. It is felt that at present there are too few films available to justify the required expenditure.

When one considers the state of present perfection to which the cinema has attained it seems strange that greater use has not been made of this most graphic means of impressing the field of consciousness. The eye makes a greater appeal than the spoken word, and propagandists are not slow to appreciate this—as witness the commencing activities of our well-meaning but misguided "Pussyfoot" cousins. But few of the many thousands who hourly witness "the pictures" can conceive of the infinite labour, the painstaking and meticulous care, bestowed by many minds, under most favourable studio conditions, before even the most modest "picture" can be produced. It is this call for concerted and purposive effort, this need for studio facilities, and the paramount necessity of intelligent production, which must limit the possibilities of the cinema as a serious help in surgical teaching. Granted, however, funds and organisation, a studio and a producer, and the collaboration of teaching bodies, much could be done, and Dr. Robinson's film might well be the first of a library of films for teaching purposes, which could be "released" in turn to teaching bodies throughout the country.

### THE DEFECTS OF ITS VIRTUES.

The method of presenting "animated diagrams," such as those exhibited at the Royal Society of Medicine, is too expensive to be practical, for it entails the execution by hand of some thousands of separate pictures. Actual cinematograph pictures are must useful, and there are many phases of clinical medicine and surgery which can be admirably presented by this means. The deformities of orthopædic practice, the gait and the motor affliction of the nervous system, the physiognomy of the insane, the methods of stretcher and field ambulance work, the intricate details of methods of femur splinting—to name but a few—would all lend themselves to graphic projection. It is a method of teaching, however, which has the defects of its virtues, and could as readily spread error as propagate truth. This was apparent in the film shown by Dr. Robinson, where no effort was made to save the perineum toward the end of the second stage of labour. It is, of course, obvious that the introduction of the accoucheur's hand in the photographic field must obscure all other processes. Such medical pictures, especially those exposing so intimate a process as labour, would require the most vigilant caution to keep them only for medical eyes.

Dr. Robinson is an enthusiast, but we fear it will be long before his vision of cinematographic teaching will be realised in this country. To be of value it would call for an organisation and unity from teaching bodies to which it is unlikely that their altruism would be equal.

